# Putting *AlphaFold* models to work with *phenix.process_predicted_model* and *ISOLDE*

**DOI:** 10.1107/S2059798322010026

**Published:** 2022-10-27

**Authors:** Robert D. Oeffner, Tristan I. Croll, Claudia Millán, Billy K. Poon, Christopher J. Schlicksup, Randy J. Read, Tom C. Terwilliger

**Affiliations:** a Department of Haematology, University of Cambridge, Cambridge Institute for Medical Research, Cambridge Biomedical Campus, The Keith Peters Building, Hills Road, Cambridge CB2 0XY, United Kingdom; b Molecular Biophysics and Integrated Bioimaging, Lawrence Berkeley National Laboratory (LBNL), Building 33R0349, Berkeley, CA 94720-8235, USA; c New Mexico Consortium, Los Alamos National Laboratory, 100 Entrada Drive, Los Alamos, NM 87544, USA; Institute of Integrative Biology, University of Liverpool, United Kingdom

**Keywords:** *AlphaFold*, *Phenix*, *ISOLDE*, *process_predicted_model*, confidence measures

## Abstract

*AlphaFold* has recently become an important tool in providing models for experimental structure determination by X-ray crystallography and cryo-EM. Large parts of the predicted models typically approach the accuracy of experimentally determined structures, although there are frequently local errors and errors in the relative orientations of domains. Importantly, residues in the model of a protein predicted by *AlphaFold* are tagged with a predicted local distance difference test score, informing users about which regions of the structure are predicted with less confidence. *AlphaFold* also produces a predicted aligned error matrix indicating its confidence in the relative positions of each pair of residues in the predicted model. The *phenix.process_predicted_model* tool downweights or removes low-confidence residues and can break a model into confidently predicted domains in preparation for molecular replacement or cryo-EM docking. These confidence metrics are further used in *ISOLDE* to weight torsion and atom–atom distance restraints, allowing the complete *AlphaFold* model to be interactively rearranged to match the docked fragments and reducing the need for the rebuilding of connecting regions.

## Abstract 2

*phenix.process_predicted_model* and *ISOLDE* provide seamless integration of *AlphaFold*-predicted models into protein structure-solution workflows.

## 1. Introduction

Until recently, the typical workflow for macromolecular crystal structure solution was roughly guided by the following protocol. Firstly, collect diffraction data and identify possible pathologies in the data. Secondly, phase the data by molecular replacement (MR) using a homologue from the Protein Data Bank (PDB; Berman *et al.*, 2003 ▸). In difficult cases, test different ways of preparing models, different homologues and ensembles of homologues. If MR fails or there are no homologues in the PDB, attempt to solve the structure using experimental phasing methods.

In this protocol, the phasing step by MR is essentially a rigid-body refinement of the model to best match the diffraction data. Suitable parts of the model may be broken into domains and refined as independent rigid bodies, improving agreement with the data if the relative orientations of the domains are incorrect. This would often be the case if the model were not a close homologue. However, breaking a model into domains when not necessary exacts a penalty since the score values for the correct placement and orientation of individual domains diminish the smaller they are. Some elaborate strategies have been developed to address this limitation. For instance, in *ARCIMBOLDO* (Millán *et al.*, 2015 ▸) the phasing is bootstrapped with *SHELXE* (Thorn & Sheldrick, 2013 ▸) using small fragments of secondary-structure elements or folds from fragment libraries. In the *AMPLE* pipeline (Bibby *et al.*, 2012 ▸), *ab initio* models produced by *ROSETTA* (Shortle *et al.*, 1998 ▸) are used to phase small structures and MR solutions are subsequently verified using *SHELXE*. Protocols such as these have inherent limitations in structure size or X-ray data resolution, and the required CPU time increases dramatically with the difficulty of the problem.

With the advent of *AlphaFold* (Jumper *et al.*, 2021 ▸) the phasing step has become far simpler in practice. Whether there are close homologues in the PDB or only, at best, distant homologues, the *AlphaFold* models will usually be of sufficient quality to solve the MR problem. Experimental phasing is becoming a niche method for the few structures where *AlphaFold* fails to provide a good model (McCoy *et al.*, 2022 ▸). Because the starting models are typically much better, partic­ularly when there are no close homologues in the PDB, and they already possess the correct sequence, the process of model building, refinement and validation is also generally much more straightforward.

A model predicted by *AlphaFold* repurposes the *B*-factor column of the PDB file for pLDDT, the predicted value of the local distance difference test (LDDT) score (Mariani *et al.*, 2013 ▸). It is therefore necessary to convert these values into the corresponding *B* factors before employing the model file in software for solving structures. Indeed, failing to convert the pLDDT values will hinder structure solution because they have an inverse relationship to *B* factors: without conversion, the highest confidence residues will be given the lowest weight in MR calculations.

For cryo-EM structure determination there is no phase problem, but the availability of *AlphaFold* models similarly greatly simplifies the process of building the initial model (by the docking of individual rigid components) and then rebuilding and refining it.

The *phenix.process_predicted_model* tool enables the easy integration of *AlphaFold* models into the structure-solution pipeline for even casual users. In the following, we describe the implementation of *phenix.process_predicted_model* and how it is integrated into the *Phenix* software suite (Liebschner *et al.*, 2019 ▸) together with *AlphaFold*. We show three examples of its use in molecular replacement. The first two examples apply two different methods to split up the predicted model into individual domains, leading to success in the MR calculation. The third example discusses a case where *phenix.process_predicted_model* trims away low-confidence regions of a predicted model that would otherwise lead to severe packing clashes in the subsequent MR calculation. In addition, we discuss how the PAE matrix may be used in *ISOLDE* in a challenging structure at lower resolution, which requires extensive rebuilding of connecting regions. Here we show how the docked MR fragments may be used as a guide for re­modelling of the complete predicted model in *ISOLDE* with the support of confidence-weighted distance and torsion restraints. This approach allows rapid modelling of the flexible connecting regions that in current standard practice would typically be traced by multiple rounds of automatic and/or manual tracing through the residual density.

## 2. Tailoring the *AlphaFold Colab* notebook to *Phenix*

The Google DeepMind software *AlphaFold* can be run with a Google login on the Google cloud computing platform with the *Colab* notebook service. The *AlphaFold* team created an *AlphaFold Colab* notebook and the *ColabFold* team created a simpler version called *ColabFold*: *AlphaFold* with *MMseqs*2 (Mirdita *et al.*, 2022 ▸). The notebook for *Phenix* is a further simplified version of the *ColabFold* notebook suitable for use with *Phenix*. Furthermore, it allows the user to include additional models as templates for *AlphaFold* when generating a new predicted model (Terwilliger *et al.*, 2022 ▸). The notebook is invoked from the *Phenix* GUI, which opens it in the default web browser on the user’s computer.

### 2.1. How *phenix.process_predicted_model* works

The *phenix.process_predicted_model* tool uses estimates of the uncertainty supplied by structure-prediction tools in the *B*-value (atomic displacement parameter) field of a model to create new pseudo-*B* values, to remove uncertain parts of the model and to break up the model into domains.

The *B*-value field in a predicted model can represent one of three possible values: an actual *B* value (atomic displacement parameter), an estimate of positional errors (r.m.s.d., provided in *RoseTTAFold*; Baek *et al.*, 2021 ▸) or the pLDDT confidence measure on a scale of either 0 to 1 or 0 to 100.

In *phenix.process_predicted_model*, positional error estimates or confidence values are used to prune the least reliable residues and are then converted to *B* values for the remaining residues. Finally, these residues are optionally grouped into domains.

## 3. Conversion of error estimates to *B* values

Positional error estimates are converted to *B* values using the standard formula (1) for the relationship between the 3D r.m.s. positional variation Δ and the corresponding *B* value,

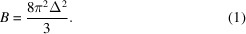




The application of this *B* value has the effect of smearing the electron density of an atom over a 3D Gaussian probability corresponding to the input r.m.s.d. (Read, 1990 ▸). We have shown previously that using such *B* values to downweight the less reliable parts of a model adds considerable value to the predicted models when used in MR, if the error estimates are reliable (Bunkóczi *et al.*, 2015 ▸; Croll *et al.*, 2019 ▸; Millán *et al.*, 2021 ▸).

### 3.1. Conversion of pLDDT values to error estimates

Because *AlphaFold* scripts can differ in whether pLDDT values are reported as a fractional or a percentage score, the *process_predicted_model* script first ensures that they are put on a fractional scale of 0 to 1. The user can explicitly specify the scale; otherwise, the scale is automatically inferred from the range of values observed.

The pLDDT values on a scale of 0 to 1 are then converted to error estimates Δ using an empirical formula (Baek *et al.*, 2021 ▸; Hiranuma *et al.*, 2021 ▸),

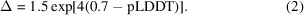




This empirical formula produces results in keeping with intuition, with estimated r.m.s. errors of nearly 25 Å (consistent with a random fold) for a pLDDT of 0 and of 0.45 Å for a pLDDT of 1, similar to the coordinate differences seen between different crystal forms of the same protein. A pLDDT value of 0.7 (which is proposed below as a default threshold for discarding low-confidence regions) corresponds to an estimated r.m.s. error of 1.5 Å.

### 3.2. Trimming away low-confidence regions from predicted models

Although considerable value for MR and docking is added to a predicted structure by downweighting regions that are expected to have only moderate errors, we find that often it is better to completely remove very low confidence regions. There are two reasons for this. Firstly, low-confidence regions are frequently in a poorly folded conformation, leading to clashes in the crystal packing. Secondly, the pLDDT and r.m.s. scores are calibrated for the positions of C^α^ atoms. When the prediction is accurate similar coordinate errors are likely to apply to other atoms in the residue, but when the prediction has low confidence the uncertainty of the local conformation increases the errors expected in other atoms. The inclusion of overweighted low-confidence residues will degrade the LLG score; at best this will slow down the calculation, but at worst the signal required to find a clear solution could be lost.

The default threshold in *phenix.process_predicted_model* is a fractional pLDDT value of 0.7, which translates to an r.m.s.d. value of 1.5 Å or to a *B* value of about 60 Å^2^. This threshold is under user control.

## 4. Splitting a trimmed model into domains

When the relative orientations of domains within a chain are uncertain, it is often useful to divide the predicted structure into separate rigid bodies that can be placed independently by molecular replacement (crystallography) or by docking (cryo-EM). Visual inspection of the portions of a chain that remain after trimming low-confidence regions can be effective in identifying compact domains, but an automated approach is helpful in a structure-determination pipeline.

Two methods are available in *phenix.process_predicted_model*. One is based on finding compact domains using only structural information, while the other is based on parsing the predicted aligned error (PAE) matrix (for *AlphaFold* models only).

### 4.1. Finding domains from a low-resolution model representation

The method used is to calculate a low-resolution map based on the input model and then to identify large blobs in that low-resolution map that are likely to correspond to domains. The low-resolution map is calculated at a resolution defined by the domain_size keyword (default 15 Å). This map is analysed to identify blobs of density. The strategy used is to find a contour level in the map that is high enough to not contain largely noise (the default is at least half the maximum density in the map) and that is low enough to have multiple regions. The low-resolution map is then contoured at varying cutoff levels, ranging from half the maximum density in the map to the maximum density in the map. For each cutoff, all contiguous regions in the map where all points in a region have a value above the cutoff are identified. The cutoff that yields the largest number of unique contiguous regions is then chosen and the corresponding contiguous regions are noted. Every point in the map is then assigned to one of these unique regions by sequentially assigning all of the points adjacent to an existing region to that region until all points are assigned. Once all regions are specified, each C^α^ atom in the *AlphaFold* model is assigned to the region in which it is located, resulting in domains that are represented as groups of segments of the *AlphaFold* model, with one domain corresponding to each region. Finally, the assignment of residues to domains is adjusted to eliminate very short segments (a default of ten residues or fewer, accomplished by moving short segments into a domain that contains neighbouring residues) and to ensure that segments that could be assigned to either of two domains are placed in the domain with the largest number of contacts.

When using this method, the recommended way to adjust the number of domains obtained is to alter the target domain size (default radius of 15 Å). Alternatively, the number can be restricted using the maximum_domains keyword (default 3).

This method works generally, not just for models predicted by *AlphaFold* but also for models derived from other sources, for example cryo-EM structures.

### 4.2. Finding domains by parsing the predicted aligned error (PAE) matrix

This method analyzes the PAE matrix provided by *AlphaFold* and finds groupings of residues that have a small mutual alignment error, which often correspond to domains.

Note that the PAE matrix is not symmetrical because entry *ij* represents the expected error in the position of residue *j* when residue *i* of the model is superimposed on the same residue in the true structure. If the local main-chain conformation of residue *i* is less certain than the local main-chain conformation of residue *j*, entry *ij* in the matrix will indicate a larger error than entry *ji*. One analogy to this is a sailor with binoculars in a boat on a wavy sea aiming the binoculars at a lighthouse on land. Whereas the sailor will often miss focusing on the lighthouse, the lighthouse keeper will easily observe the sailor in the boat bobbing up and down on the waves. The lower of these two entries is a better indication of how well their relative position in space is known, so the PAE matrix is pre-processed by setting all pairs of off-diagonal entries to the lower of the two values.

Identification of residue groupings with low mutual error is performed by carrying out a community clustering analysis of the pre-processed PAE matrix. Each residue is treated as a node in a graph, and an edge is formed between each pair of residues with a mutual PAE below a cutoff of *c* (typically *c* = 5 Å); the edge is given a weight of (PAE)^−*p*^, where typically *p* = 1. The cutoff *c* and weight exponent *p* are user-adjustable via the pae_cutoff and pae_power arguments, respectively; optionally, edges may be further weighted according to the distance between C^α^ atoms. The graph is then partitioned using the Clauset–Newman–Moore greedy modularity maximization algorithm implemented in *NetworkX* (Hagberg *et al.*, 2008 ▸; Clauset *et al.*, 2004 ▸).

In *Phenix* 1.20, the recommended way to adjust the number of domains found is to change the value of pae_power, with larger values leading to more domains. In the upcoming Python 3 release of *Phenix* it will be possible to further fine-tune the stringency of the clustering via the graph_resolution argument (discussed in the documentation of the implementation of *NetworkX*), with higher values giving a larger number of domains. The result can be restricted to only the few largest domains using the maximum_domains keyword as noted above.

### 4.3. Implications of domain size and number for molecular replacement

Chances of success in MR can be judged by the value expected for the LLG (eLLG), which can be evaluated from the data quality and extent, the fraction of the asymmetric unit accounted for by the model and the effective r.m.s. error predicted for the model (Oeffner *et al.*, 2018 ▸). If a multi-domain model has substantial domain motions relative to the target structure, the effective r.m.s. error for the whole model will be much larger than for the individual domains. In such a case, it is essential to divide the model into separate rigid domains, which will yield higher LLG values. Given that the eLLG depends on the square of the model completeness, there is a limit (dependent on data resolution) on how small the individual domains can be and still give significant signal in the MR search. Accordingly, an advisory is given in *Phaser* (McCoy *et al.*, 2007 ▸) prior to the MR calculation informing the user whether the search components are of adequate size for a successful first placement as well as subsequent placements.

The eLLG calculation, and the strategies deduced from it, depend on the r.m.s. error assigned to the model. Before the advent of *AlphaFold*, the required coordinate error for a model obtained from a homologue would be derived primarily from the sequence identity to the unknown structure (Hatti *et al.*, 2020 ▸). Although we have not yet undertaken a similar comprehensive study of the optimal predicted coordinate error for *AlphaFold* models, experience so far with dozens of models prepared with *process_predicted_model* suggest that a value of 1 Å is a reasonable starting estimate.

Note that if *process_predicted_model* suggests that an *AlphaFold* model should be divided into domains, but *Phaser* then predicts that structure solution will be very difficult, it can be productive to test the possibility that the relative domain orientations may indeed be correct by including larger models among those that are tested in MR.

## 5. Using *phenix.process_predicted_model*

A typical command-line invocation of *phenix.process_predicted_model* is as follows: phenix.process_predicted_model my_model.pdb b_value_field_is=lddt pae_file=my_pae.json.

This will convert the *B*-value field in my_model.pdb from pLDDT scores to *B* values, trim residues with pLDDT less than 0.7 and write out a new model with individual chains (separate chain ID values) corresponding to domains identified from the PAE matrix in my_pae.json. A listing of other optional arguments can be seen via the command phenix.process_predicted_model --show-defaults.

The *phenix.process_predicted_model* tool can also be accessed using the *Phenix* GUI. Using the default values may or may not generate the desired number of domains. In such cases it is advisable to try nondefault values of the parameters as described in Sections 4.1 and 4.2.

Note that for multimer predictions domain parsing using the PAE matrix is not available, and only one chain can be processed at a time.

## 6. Confidence-weighted distance and torsion restraints in *ISOLDE*

**Figure 1:**
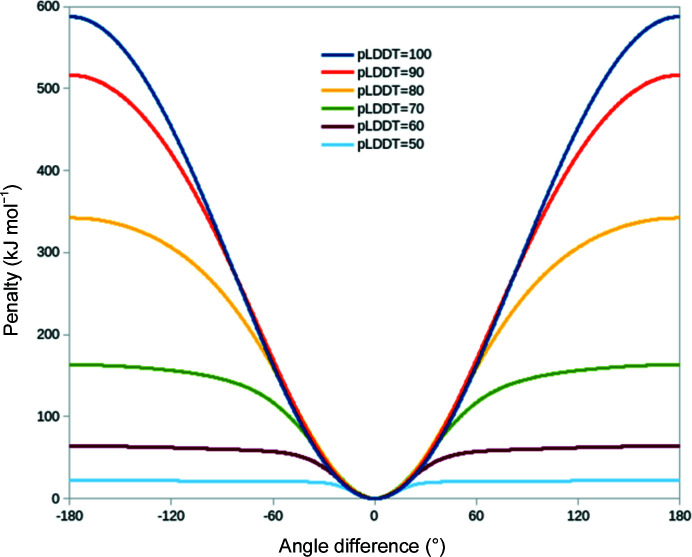
Default weighting of reference-model torsion restraints in *ISOLDE* according to pLDDT. The φ, ψ and (if the template and model residues have the same identity) every χ dihedral in a given residue are restrained based on the pLDDT for the matching template residue; the ω dihedral is restrained to *cis* or *trans* ± 30° using the flat-bottom torsion restraints in *ISOLDE*. Residues where the template pLDDT is less than 50 are not restrained.

**Figure 2:**
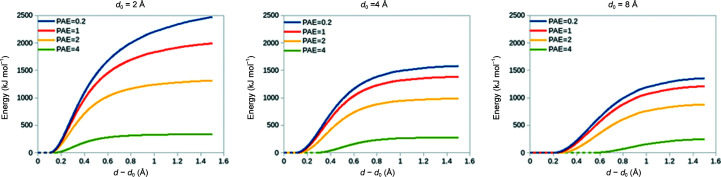
Default weighting of reference-model distance restraints in *ISOLDE* according to min[PAE(*i*, *j*), PAE(*j*, *i*)]. Here *d*_0_ is the distance in the template between a given restrained atom pair and *d* is the current instantaneous distance in the working model. No restraints are formed between pairs of residues with mutual PAE values greater than 4 Å. The most important outcome of this is that it avoids the introduction of spurious restraints in the somewhat common scenario where *AlphaFold* places domains with no real-world interaction in close proximity due to random chance.

It is well established that when the starting model is significantly out of step with the experimentally determined map some form of conformational restraint is required to prevent severe deformation during gradient-driven flexible fitting (Trabuco *et al.*, 2009 ▸), where each heavy atom is simply biased towards the nearest region of high density. This is particularly true in the crystallographic environment, where severely displaced parts of the model often overlap with strong density from symmetry neighbours. However, it is generally in­advisable to over-restrain the model, since this would prevent successful fitting of sites where the experimental conformation is slightly different from the otherwise correctly placed model. As described previously (Croll & Read, 2021 ▸), *ISOLDE* implements distance and torsion restraint schemes based on an ‘adaptive’ loss function that was originally derived for use in machine learning (Barron, 2019 ▸). While these schemes were designed to support encoding of confidence (on a per-restraint basis) in the strength, width and rate of fall-off of the applied bias, prior to the advent of *AlphaFold* this flexibility went largely unused. Since version 1.3, *ISOLDE* has made use of *AlphaFold* pLDDT values to adjust each torsion restraint, and residue–residue PAE values to adjust distance restraints (and exclude low-confidence distances entirely). The current forms of these restraints are shown in Fig. 1 ▸ for torsions and in Fig. 2 ▸ for distances. Restraints may be applied via the isolde restrain torsions and isolde restrain distances commands using the argument adjustForConfidence true. In *ISOLDE* 1.3 confidence weighting of distance restraints was only available for precalculated predictions fetched from the *AlphaFold* Protein Structure Database; *ISOLDE* 1.4 supports the use of any user-supplied *AlphaFold* prediction with its associated PAE matrix.

For a given residue, all φ, ψ and χ dihedrals are given the same weight and confidence terms. While in principle it may be preferable to downweight restraints on outer χ dihedrals, in practice we find that for the majority of high-confidence residues, in particular those forming the packed core of the protein, the predicted side-chain geometry is correct for all dihedrals. As such, a blanket downweighting of side-chain dihedral restraints would probably be counterproductive.

## 7. Worked examples

### 7.1. Worked example 1: molecular replacement with domains derived from the PAE matrix

As the first test case, we chose PDB entry 6l5l, the crystal structure of human DExD-box RNA helicase DDX21 in the apo state (Chen *et al.*, 2020 ▸). This structure was released on 17 June 2020 and therefore was not present in the *AlphaFold* training data set, which is based on structures from the PDB up to 30 April 2018. The data contain 7209 reflections up to a resolution of 3.1 Å.

**Figure 3:**
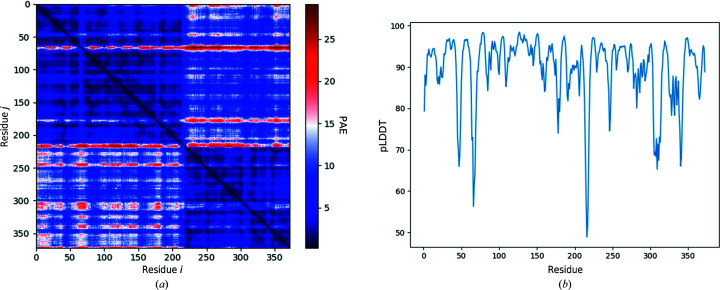
Visual representation of the uncertainty measures provided by the *Phenix Colab* notebook for the structure predicted by *AlphaFold* for the amino-acid sequence of PDB entry 6l5l. (*a*) PAE matrix, coloured from blue to red for low to high predicted aligned errors. (*b*) pLDDT (percentage scale) as a function of residue number.

The sequence of 372 residues was submitted to *AlphaFold* (through the *Phenix Colab* notebook discussed above), turning off the option to use templates from the PDB. The associated pLDDT scores per residue and PAE matrix are illustrated in Fig. 3 ▸.

**Figure 4:**
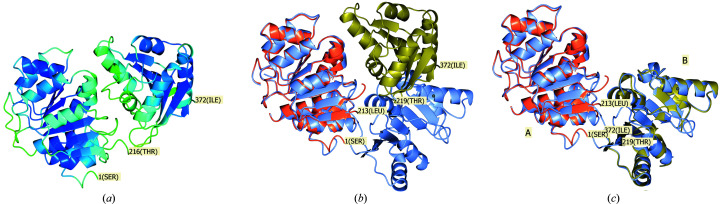
(*a*) The unprocessed structure of human DExD-box RNA helicase DDX21, predicted by *AlphaFold*, coloured by the pLDDT value in the *B*-factor field of the PDB file. The colouring varies smoothly from blue through green to red, corresponding to pLDDT values of 100, 80 and 0, respectively. The N- and C-termini (residues 1 and 372) are labelled. Residue 216 has been labelled as a potential boundary between rigid domains found by visual inspection of the molecule. (*b*) The processed predicted structure superposed onto the deposited target structure, PDB entry 6l5l, in grey-blue using secondary-structure matching (*SSM*; Krissinel & Henrick, 2004 ▸). (*c*) The successful MR solution of the processed and predicted structure divided into two chains stripped of residues with pLDDT less than 70. The MR solution is superposed on the target structure with *phenix.find_alt_orig_sym_mate*, accounting for allowed symmetry and origin shifts. Figures were produced with *CCP*4*mg* version 2.10 (McNicholas *et al.*, 2011 ▸).

The visual representation of the PAE matrix is based on the implementation by the *ColabFold* team (Mirdita *et al.*, 2022 ▸). The predicted model together with the PAE matrix was used as input for *phenix.process_predicted_model* to partition the model into separate domains, as illustrated in Fig. 4 ▸(*b*). With default parameters, the structure-based domain-identification algorithm (which does not use the PAE matrix) fails to identify these domains, although we did not investigate whether it might succeed with some nondefault parameters.

In Fig. 4 ▸(*a*) we note that residue Thr216 links potential compact domains. This residue number is also near the boundaries between two dark blue squares along the diagonal of the PAE matrix in Fig. 3 ▸(*a*) as well as yielding the minimum pLDDT value in Fig. 3 ▸(*b*). These observations support the finding that the residues around Thr216 form a domain boundary.

After processing the *AlphaFold* model, the pLDDT values in the *B*-factor column of the new model have been converted to pseudo-*B* factors, enabling the use of the processed model for subsequent structure-solution programs.

#### 7.1.1. Testing the models predicting PDB entry 6l5l in molecular-replacement calculations

MR calculations on the processed model predicting the structure of PDB entry 6l5l were performed with *Phaser* (McCoy *et al.*, 2007 ▸). The hardware used was a Windows 10, 64-bit desktop PC with an Intel Xeon CPU with eight cores (3 GHz) and 32 GB memory. The first calculation was performed with the predicted model as one single ensemble not divided into individual domains. The MR calculation terminated unsuccessfully.

For the second calculation, the two chains produced by *phenix.process_predicted_model* as in Fig. 4 ▸(*c*) were used as two separate search ensembles for the MR calculation. The MR calculation succeeded in placing both components with an LLG value of 567 and a TFZ score of 21. In both cases a generic estimated r.m.s. error of Δ = 1.0 Å was used together with the deposited sequence and reflection data file. In the above calculation case *Phaser* refines the input Δ values to r.m.s. values of 1.02 and 0.92 Å for the two search components chains *A* and *B*, respectively. This adds support to our informal conclusion from other tests that Δ = 1.0 Å is an appropriate generic input value.

The superposition of the two-chain MR solution on top of the deposited crystal structure using *phenix.find_alt_orig_sym_mate* (Oeffner *et al.*, 2012 ▸) is shown in Fig. 4 ▸(*c*). Both search components superpose well onto the target structure, with *SSM* reporting r.m.s.d. values to the deposited target structure of 0.83 and 1.16 Å for chains *A* and *B*, respectively.

### 7.2. Worked example 2: molecular replacement with domains derived from low-resolution blobs

As a second test case, we chose PDB entry 6j09, the crystal structure of *Haemophilus influenzae* BamA (Ma *et al.*, 2019 ▸). This structure was released on 30 October 2019 and therefore also was not present in the *AlphaFold* training data. As the structure was solved by MR based on a structure released in 2007 one might anticipate that the predicted structure would be largely correct, but internal domain movements complicate this. The data contain 12 972 reflections up to a resolution of 3.0 Å.

**Figure 5:**
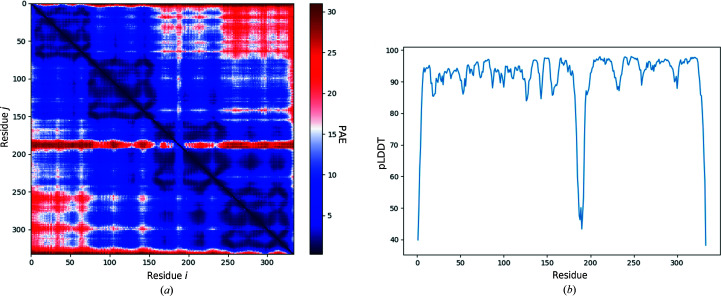
Visual representation of the uncertainty measures provided by the *Phenix Colab* notebook for the structure predicted by *AlphaFold* for the amino-acid sequence of PDB entry 6j09. (*a*) PAE matrix, coloured from blue to red for low to high predicted aligned errors. (*b*) pLDDT (percentage scale) as a function of residue number.

The sequence of 333 residues was submitted to *AlphaFold* (through the *Phenix Colab* notebook discussed above). The associated pLDDT scores per residue and PAE matrix are illustrated in Fig. 5 ▸.

Using a nondefault value of domain_size=19.0, *phenix.process_predicted_model* split the predicted model into three chains. The PAE matrix illustrated in Fig. 5 ▸(*a*) is visually suggestive of the existence of three or four domains. However, when *phenix.process_predicted_model* was provided with this PAE matrix it did not divide the model into separate chains. As in the previous example, we have not investigated whether some set of nondefault parameters would produce three chains when using the PAE matrix in *phenix.process_predicted_model*.

#### 7.2.1. Testing the models predicting PDB entry 6j09 in molecular-replacement calculations

**Figure 6:**
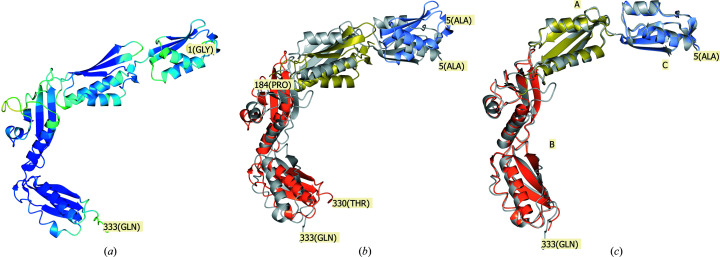
(*a*) The unprocessed predicted structure of PDB entry 6j09 coloured by pLDDT. (*b*) The processed predicted structure split into three domains, coloured coral, gold and ice blue, superposed with *SSM* as one rigid model onto the target structure in grey. (*c*) MR solution using the three domains obtained from *phenix.process_predicted_model* as search components and superposed onto the target structure with *phenix.find_alt_orig_sym_mate*. Figures were produced with *CCP*4*mg*.

The first calculation was performed on the processed model retained as one rigid search model, as illustrated in Fig. 6 ▸. The MR solution was incorrect with correspondingly poor scores (LLG = 24.6, TFZ = 6.2). The *SSM* superposition in Fig. 6 ▸(*b*) shows that internal domain motions effectively prevent a perfect superposition, and consequently a good MR solution, when using the processed model as one rigid search model.

A second calculation was then performed using the processed model split into three separate domains without using the PAE matrix, with the result illustrated in Fig. 6 ▸(*c*). The MR calculation succeeded with high scores (LLG = 1013.2, TFZ = 30.7 for the last domain placed). The r.m.s. values for chains *A*, *B* and *C* in Fig. 6 ▸(*c*) were refined to 0.26, 1.72 and 0.59 Å, respectively. The r.m.s.d. values reported by *SSM* between the MR solution and the target structure are 0.63, 1.04 and 0.69 Å for chains *A*, *B* and *C*, respectively.

When the number of reflections is sufficiently large in a data set relative to the number of atoms, *Phaser* frequently succeeds in finding a solution that places one or more domains, but not all, correctly. A subsequent occupancy refinement (triggered by detecting bad clashes for a solution with a high TFZ score that indicates confidence) will, in favourable cases, assign zero occupancy to incorrectly placed domains or residues in the rigid model that would otherwise clash with symmetry copies during packing of the unit cell. In the above study, because of the low resolution of these two data sets, partially correct placements do not yield a clear MR search signal and the search models must be partitioned into separate domains to obtain correct MR solutions.

### 7.3. Worked example 3: molecular replacement using a model with low-confidence regions

The crystal structure of human exonuclease 5 (PDB entry 7lw7; Hambarde *et al.*, 2021 ▸) illustrates the issues encountered with low-confidence regions and the advantages of deleting them. This structure was released after *AlphaFold* training had taken place. In the *AlphaFold* Protein Structure Database (Varadi *et al.*, 2022 ▸; Jumper *et al.*, 2021 ▸), parts of the backbone of the crystallized protein such as from Leu31 to Leu70 and from Gly357 to Lys373 have been predicted to have low LDDT values (see Section S1). These parts can optionally be retained in the output model from *phenix.process_predicted_model*. However, an MR calculation using the model retaining the low-confidence residues rejects the correct solution due to packing clashes. On the other hand, an MR calculation using a model in which *phenix.process_predicted_model* has stripped away low-confidence regions trivially finds the correct solution, with a higher LLG score (676) than the rejected solution using the untrimmed model (582).

**Figure 7:**
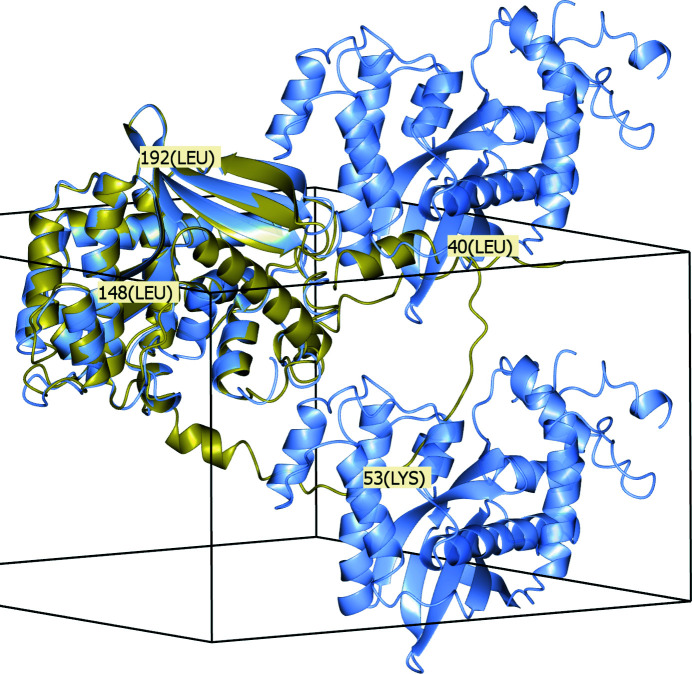
The untrimmed predicted model (gold) superposed onto the crystal structure for PDB entry 7lw7 (ice blue) including two symmetry copies. This figure was produced with *CCP*4*mg*.

In Fig. 7 ▸ the untrimmed predicted model for PDB entry 7lw7 is shown superposed with *SSM* onto the target structure in the crystal unit cell together with two symmetry copies. It is evident that the untrimmed model cannot render an MR solution. For instance, residues around Lys53 and Leu40 of the untrimmed model overlap with symmetry copies of residues around Leu148 and Leu192, respectively.

### 7.4. Worked example 4: a lower resolution data set requiring extensive rebuilding

**Figure 8:**
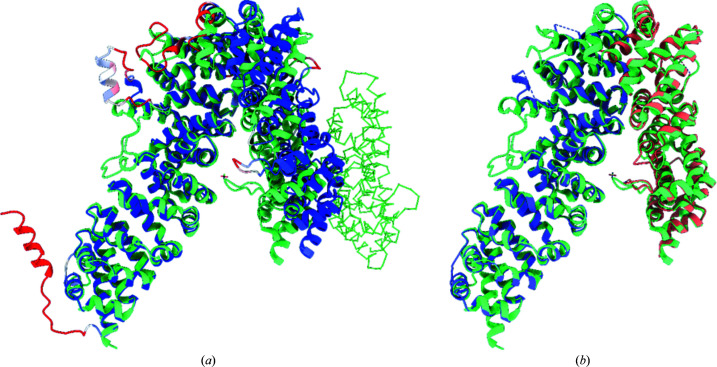
(*a*) PDB entry 3now (light green) adopts a V-shaped conformation. The processed *AlphaFold* prediction (coloured by *B* factor; blue, 0 Å^2^; red, ≥100 Å^2^) has a significantly wider hinge angle, causing a severe clash of the N-terminal domain with a symmetry mate (green C^α^ trace). (*b*) Using two domains identified from the PAE matrix by *phenix.process_predicted_model* (purple, orange) yields a strong MR solution.

As a more challenging example, we chose PDB entry 3now, an 810-residue, 2.99 Å resolution structure of UNC-45 from *Drosophila melanogaster* (Fig. 8 ▸; Lee *et al.*, 2011 ▸). UNC-45 forms a lopsided inverted V, with the N-terminus forming the shorter arm. The *AlphaFold* model is locally quite accurate, but has a large difference in the relative positions of domains from the deposited structure approximately perpendicular to the plane of the V, leaving the N-terminus offset by about 15 Å when the C-terminal domain is aligned with the deposited structure. While naïve use of the *AlphaFold* model (after trimming low-confidence residues and converting pLDDT scores to *B* factors) in *Phaser* leads to a solution (LLG = 163, TFZ = 17.6) with the C-terminal domain correctly placed, the N-terminal domain clashes severely with its symmetry equivalent and is automatically reduced to zero occupancy by *Phaser*. Using the PAE matrix splits the model into two domains encompassing 692 residues (compared with 786 residues modelled in the deposited structure) which give a much higher quality solution (Fig. 8 ▸ *b*; LLG = 2331, TFZ = 47).

**Figure 9:**
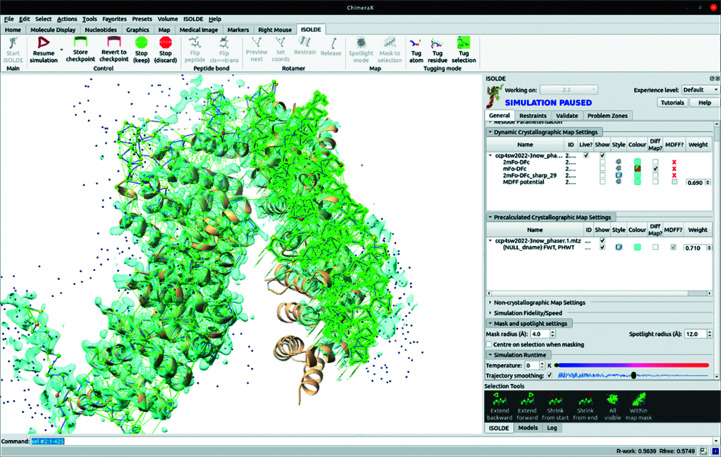
Interactive refitting of the complete model in *ISOLDE*. The MR solution is shown as a tan ribbon; the complete model is initialized in *ISOLDE* and shown as a C^α^ trace. The region highlighted in bright green is selected and is being pulled by the ‘tug selection’ mouse mode in *ISOLDE*, with distances and torsions restrained to the *AlphaFold* model geometry. For this initial step the map generated by *Phaser* is used as the fitting potential; once the gross rearrangement is complete further rebuilding will use structure factors calculated on-the-fly from the model.

While this solution may be used as the basis for standard model-completion algorithms, the generally high quality of the complete *AlphaFold* prediction allows a potentially faster approach which we explored here (Fig. 9 ▸ and Supplementary Movie S1). In brief, the MR solution was only used to provide (i) a target guide and (ii) a preliminary map for interactive refitting of the complete model in *ISOLDE*. The model was first restrained using the isolde restrain distances and isolde restrain torsions commands as described in Section 6, and maps were generated from the *Phaser* output MTZ file. In standard *ISOLDE* runs the precalculated maps would typically be ignored in favour of an MDFF potential calculated ‘live’ based on the current model coordinates. Here, we temporarily disabled this and instead enabled the *Phaser* 2*mF*_o_ − *DF*_c_ map. Then, in an interactive simulation the out-of-position N-terminal domain (approximately residues 140–420) was selected and pulled into position with the ‘tug selection’ right mouse mode of *ISOLDE*. On our test machine (a dual Xeon E5-2687W workstation equipped with an Nvidia Titan Xp GPU), this initial ‘gross’ refitting simulation took a little over 1 min and was accompanied by a reduction in *R* factor from 0.57 to 0.41. At this point the precalculated *Phaser* map was discarded, and all further rebuilding was performed into the maps calculated live by *ISOLDE*.

**Figure 10:**
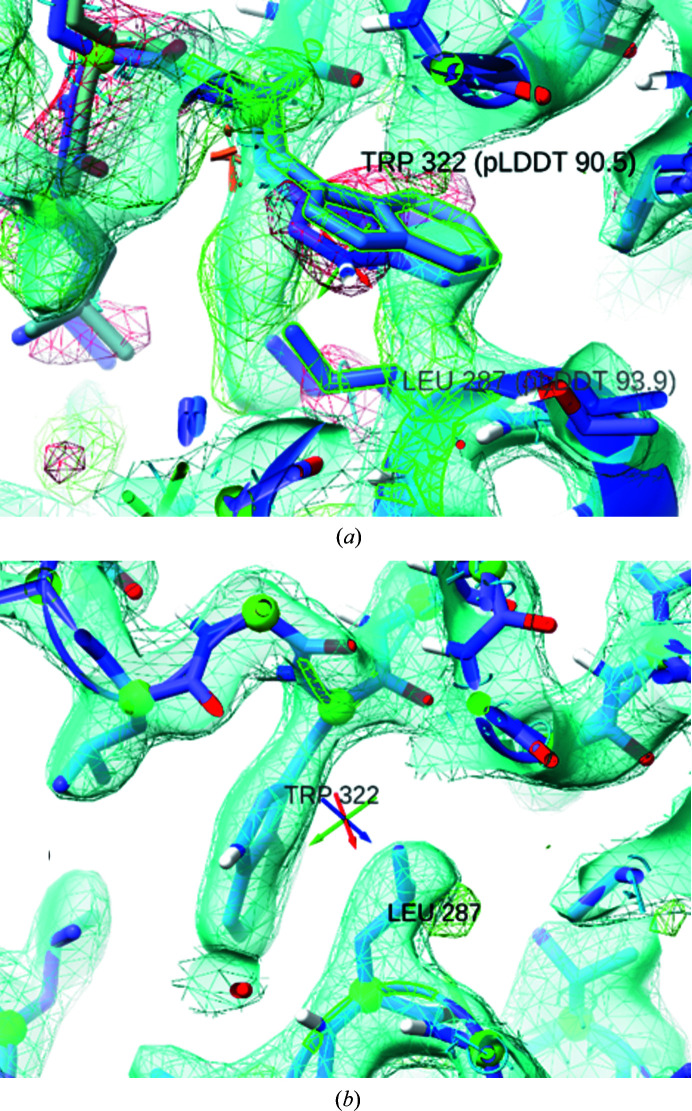
High pLDDT does not always indicate local correctness. (*a*) Despite very high pLDDT scores of 93.9 and 90.5, respectively, Leu297 and Trp322 were predicted with incorrect rotamers. The loop following Trp322 was also badly wrong (albeit at significantly lower confidence). (*b*) The region may be straightforwardly rebuilt in *ISOLDE* after selectively releasing the local reference restraints. In both panels, maps were calculated on-the-fly by *ISOLDE*. Cyan wireframe, 2*mF*_o_ − *DF*_c_ at 1.5σ; cyan surface, 2*mF*_o_ − *DF*_c_ (*B*_sharp_ = 30 Å^2^) at 2σ; green and red wireframe, *mF*_o_ − *DF*_c_ at +3σ and −3σ, respectively.

It must be emphasized that the generally very high local quality of *AlphaFold* models does not absolve the user of the need to carefully check the model against the experimental density. While the majority of issues requiring rebuilding arose around symmetry interfaces, some high-confidence residues far from the site of conformational change nevertheless showed severe deviation from the map, for example Leu287 and Trp322 (Fig. 10 ▸), which stacked against each other from the wrong side; it is tempting to speculate that this contributed to the deviation of the loop C-terminal to Trp322. Errors such as these can be addressed in *ISOLDE* by selectively releasing the local reference restraints followed by interactive rebuilding.

**Figure 11:**
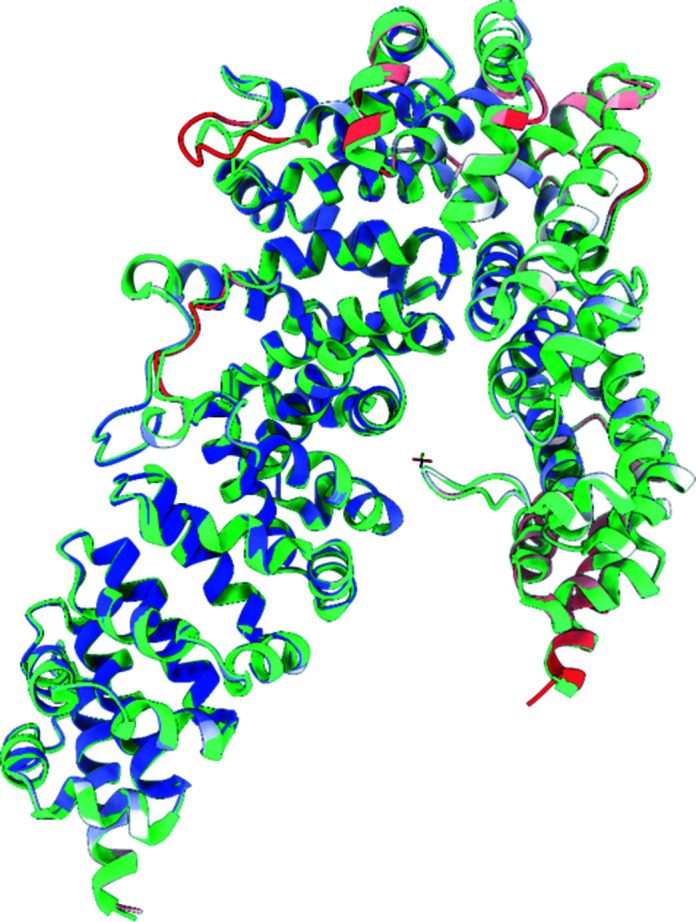
Final model after two rounds of rebuilding in *ISOLDE* with refinement in *phenix.refine* (coloured by *B* factor: blue, 35 Å^2^; red, ≥135 Å^2^) overlaid with the original PDB entry 3now.

Rebuilding the model from this point was generally quite straightforward, with the exception of the 590–620 loop (top left of Fig. 8 ▸ *a*), which undergoes a ∼30 Å shift downwards to stack in a symmetry interface and overlapped severely with a symmetry contact in the initial model. While remodelling this directly from the starting model was tractable (if challenging) in *ISOLDE*, an equally valid option may have been to initially delete this loop and to rebuild the rest of the model after refinement. An initial pass in *ISOLDE* focused on rebuilding local regions showing major errors (symmetry clashes and large deviations from the map); after refinement of the result in *phenix.refine* we undertook a second end-to-end inspection and rebuild followed by a final refinement. In total the process took about half a working day; the resulting model contained one more residue than the original, displayed significantly improved geometry and refined with *R* factors that were comparable to or slightly better than the original (Table 1 ▸). An overlay of the final model with the original is shown in Fig. 11 ▸. We note that at this stage the map showed numerous opportunities for the addition of ordered solvent molecules; while we placed six waters, complete coverage is beyond the scope of this manuscript.

## 8. Discussion

Segmenting a search model into independent rigid fragments suitable for MR has historically been somewhat challenging to automate, and was often left to the intuition of the individual crystallographer. Additionally, the use of distant homologues for phasing was generally challenging, often requiring many trials of different levels of model truncation to find a viable solution. Since the advent of *AlphaFold* the second challenge has largely been removed: after decomposition into domains, typically only the removal of the most flexible and/or uncertain loops and tails is necessary for successful phasing.

The *phenix.process_predicted_model* tool provides a keystone in automating the decomposition of a model into domains. It is versatile and can be applied to split a model into structural domains regardless of how the model has been derived. If it is the result of a prediction from *AlphaFold* it can use the associated PAE matrix, whereas if it is a model derived from a different prediction algorithm or from experiments such as X-ray or cryo-EM it will use information from the model itself to split it into domains. Depending on the anticipated size or distance between domains in a model one or the other method may be preferable for a given model in a specific circumstance. In any case, automated decomposition into domains usually yields fragments that perform well in MR. This allows integration into macromolecular structure-solution pipelines.

In all but the most trivial cases the rigid fragments suitable for MR will not correspond to the entire structure. After successful MR the typical current approach is to retrace the missing residues into the residual density, typically taking many rebuild/refine cycles to reach convergence. Modern structure predictions typically have excellent local geometry, differing only from the experimental structure in large-scale domain positioning, the disposition of loops and tails, and the occasional rotamer error. This allows a potentially far more time- and energy-efficient approach, which we have explored here: using the MR solution combined with confidence-weighted restraints to guide the matching portions of the complete model into the docked positions, allowing much of the remaining structure to settle naturally. In the current *ISOLDE* implementation this allowed a ‘from scratch’ recapitulation of PDB entry 3now in well under a day on a single workstation.

The two proteins used in the examples in Sections 7.1 and 7.2 did not form part of the training set for *AlphaFold*, although the MR models used to initially solve them did. However, as *AlphaFold* implements no hard-coded correspondence between protein sequences and the resulting protein structures and because the option to use related structures as templates was turned off, these structures are useful for testing the predictive power of *AlphaFold* and the suitability of predicted models for subsequent downstream structure-solution programs.

The development of AI predictions of protein structures is currently a vibrant field of research and we anticipate that the default values of the parameters supplied to *phenix.process_predicted_model* may change over time.

## Supplementary Material

Details of the Supporting Information files. DOI: 10.1107/S2059798322010026/ai5009sup1.pdf

The Phenix AlphaFold Colab notebook predicted structure of PDB entry 6j09. DOI: 10.1107/S2059798322010026/ai5009sup2.pdb

PAE matrix as a text file, 6J09_1_cycle_1_PAE_cycle_1.jsn, produced by Phenix AlphaFold Colab notebook as described in Section S2. DOI: 10.1107/S2059798322010026/ai5009sup3.txt

The Phenix AlphaFold Colab notebook predicted structure of PDB entry 6l5l. DOI: 10.1107/S2059798322010026/ai5009sup4.pdb

Plain text file, phenix6L5L_16_1_cycle_1_PAE_cycle_1.jsn, produced by Phenix AlphaFold Colab notebook as described in Section S2. DOI: 10.1107/S2059798322010026/ai5009sup5.txt

AF-Q9H790-F1-model_v2.pdb, the AlphaFold predicted structure of UniProt ID Q9H790 corresponding to PDB entry 7lw7. DOI: 10.1107/S2059798322010026/ai5009sup6.pdb

AF-Q9H790-F1-predicted_aligned_error_v2.json, PAE matrix of the AlphaFold prediction of UniProt ID Q9H790 corresponding to PDB entry 7lw7. DOI: 10.1107/S2059798322010026/ai5009sup7.txt

Supplementary Movie S1. DOI: 10.1107/S2059798322010026/ai5009sup8.mp4

## Figures and Tables

**Table 1:** Comparison of model statistics for the original and recapitulated PDB entry 3now Note that the new model was built with a different set of free reflections, so the *R* factors are not directly comparable.

	Original	Revised
Resolution range	49.2–2.99 (49.2–6.44)	49.2–2.99 (49.2–7.03)
Reflections used in refinement	39320 (3335)	39335 (2509)
Reflections used for *R*_free_	1865 (153)	1883 (110)
*R*_work_	0.1923 (0.2901)	0.1967 (0.2943)
*R*_free_	0.2256 (0.3351)	0.2151 (0.3239)
No. of non-H atoms	6077	6094
Protein residues	786	787
R.m.s.d., bond lengths (Å)	0.013	0.007
R.m.s.d., angles (°)	1.42	0.94
Ramachandran favoured (%)	91.58	97.83
Ramachandran allowed (%)	7.53	2.17
Ramachandran outliers (%)	0.89	0.00
Ramachandran *Z*-score	−4.62	−1.38
Rotamer outliers (%)	4.84	0.30
Clashscore	18.44	0.16
