# Supplementary material for: Putting AlphaFold models to work with phenix.process_predicted_model and ISOLDE
Source: Acta Crystallogr D Struct Biol. 2022 Oct 27;78(Pt 11):1303–14. doi: 10.1107/S2059798322010026 (PMC9629492; doi:10.1107/S2059798322010026)
Supplement: Supplementary file 1 [file d-78-01303-sup1.pdf]

STRUCTURAL
BIOLOGY

Volume 78 (2022)

Supporting information for article:

Putting *AlphaFold* models to work with *phenix.process_predicted_model* and *ISOLDE*

Robert D. Oeffner, Tristan I. Croll, Claudia Millán, Billy K. Poon, Christopher J. Schlicksup, Randy J. Read and Tom C. Terwilliger

S1. *AlphaFold* prediction of PDB ID: 7LW7

In section 8 we remark on some of the low confidence areas of the *AlphaFold* prediction of the UniProt id: Q9H790 corresponding to the PDB ID:7LW7. The *AlphaFold* Protein Structure Database contains structural predictions of the human proteome. In anticipation of future improvements to *AlphaFold* the structures in that database may not remain static over time. The *AlphaFold* prediction of the UniProt id: Q9H790 corresponding to PDB ID:7LW7 and the associated PAE matrix at the time of writing is therefore made available as supplementary material.

S2. *AlphaFold* prediction of PDB ID: 6L5L and 6j09

The *PHENIX* Colab notebook *AlphaFold* predictions of the amino acid sequence corresponding to the structures with PDB ID: 6L5L and 6j09 discussed in section 7 produces coordinate files (.pdb) and associated PAE matrix files (.jsn). All files are available as supplementary material.

S3. Additional video

The *ChimeraX ISOLDE* video mentioned in section 7.4 is available as supplementary material.
